# Combinatorial Use of Machine Learning and Logistic Regression for Predicting Carotid Plaque Risk Among 5.4 Million Adults With Fatty Liver Disease Receiving Health Check-Ups: Population-Based Cross-Sectional Study

**DOI:** 10.2196/47095

**Published:** 2023-09-07

**Authors:** Yuhan Deng, Yuan Ma, Jingzhu Fu, Xiaona Wang, Canqing Yu, Jun Lv, Sailimai Man, Bo Wang, Liming Li

**Affiliations:** 1 Chongqing Research Institute of Big Data Peking University Chongqing China; 2 Meinian Institute of Health Beijing China; 3 School of Population Medicine and Public Health, Chinese Academy of Medical Sciences & Peking Union Medical College Beijing China; 4 Department of Epidemiology and Biostatistics, School of Public Health Peking University Beijing China; 5 Peking University Health Science Center Meinian Public Health Institute Beijing China; 6 Key Laboratory of Epidemiology of Major Diseases (Peking University), Ministry of Education Beijing China; 7 MJ Health Screening Center Beijing China; 8 Peking University Center for Public Health and Epidemic Preparedness & Response Beijing China

**Keywords:** machine learning, carotid plaque, health check-up, prediction, fatty liver, risk assessment, risk stratification, cardiovascular, logistic regression

## Abstract

**Background:**

Carotid plaque can progress into stroke, myocardial infarction, etc, which are major global causes of death. Evidence shows a significant increase in carotid plaque incidence among patients with fatty liver disease. However, unlike the high detection rate of fatty liver disease, screening for carotid plaque in the asymptomatic population is not yet prevalent due to cost-effectiveness reasons, resulting in a large number of patients with undetected carotid plaques, especially among those with fatty liver disease.

**Objective:**

This study aimed to combine the advantages of machine learning (ML) and logistic regression to develop a straightforward prediction model among the population with fatty liver disease to identify individuals at risk of carotid plaque.

**Methods:**

Our study included 5,420,640 participants with fatty liver from Meinian Health Care Center. We used random forest, elastic net (EN), and extreme gradient boosting ML algorithms to select important features from potential predictors. Features acknowledged by all 3 models were enrolled in logistic regression analysis to develop a carotid plaque prediction model. Model performance was evaluated based on the area under the receiver operating characteristic curve, calibration curve, Brier score, and decision curve analysis both in a randomly split internal validation data set, and an external validation data set comprising 32,682 participants from MJ Health Check-up Center. Risk cutoff points for carotid plaque were determined based on the Youden index, predicted probability distribution, and prevalence rate of the internal validation data set to classify participants into high-, intermediate-, and low-risk groups. This risk classification was further validated in the external validation data set.

**Results:**

Among the participants, 26.23% (1,421,970/5,420,640) were diagnosed with carotid plaque in the development data set, and 21.64% (7074/32,682) were diagnosed in the external validation data set. A total of 6 features, including age, systolic blood pressure, low-density lipoprotein cholesterol (LDL-C), total cholesterol, fasting blood glucose, and hepatic steatosis index (HSI) were collectively selected by all 3 ML models out of 27 predictors. After eliminating the issue of collinearity between features, the logistic regression model established with the 5 independent predictors reached an area under the curve of 0.831 in the internal validation data set and 0.801 in the external validation data set, and showed good calibration capability graphically. Its predictive performance was comprehensively competitive compared with the single use of either logistic regression or ML algorithms. Optimal predicted probability cutoff points of 25% and 65% were determined for classifying individuals into low-, intermediate-, and high-risk categories for carotid plaque.

**Conclusions:**

The combination of ML and logistic regression yielded a practical carotid plaque prediction model, and was of great public health implications in the early identification and risk assessment of carotid plaque among individuals with fatty liver.

## Introduction

Carotid plaque is an independent risk factor for cerebral stroke [1], myocardial infarction [2], and atherosclerotic cardiovascular disease [2], which are all leading causes of death and disability worldwide [3,4], presenting severe economic burden in both developed and developing countries [5]. Nearly 20% of stroke cases were caused by carotid atherosclerotic plaque [6]. The rupture or shedding of carotid plaque can lead to thrombosis and has become the major cause of cerebrovascular accidents [3,7]. It has been proposed that nearly one-third of Chinese adults were experiencing from carotid plaque [8]. With the growing aging population and the acceleration in urbanization, the incidence rates of cardiovascular disease in China would increase steadily in the next few decades [9]. Thus, early detection of carotid plaque can bring great benefits in the timely and active prevention of stroke and other cerebrovascular and cardiovascular diseases. It is necessary to develop effective tools to identify carotid plaque in the asymptomatic population and curb its progression at an early stage.

Recently, several studies have demonstrated significant associations exist between fatty liver disease and coronary artery disease, including carotid plaque and carotid stenosis [10-12]. Individuals with fatty liver disease were proved to have an elevated risk of developing carotid plaque. However, although liver ultrasound has been incorporated into the routine check-up program, carotid artery ultrasound examination is not prevalent due to cost-effectiveness reasons for the asymptomatic population [13]. As the most widely used method for evaluating carotid plaque [14], the low prevalence of carotid ultrasound may result in missed detection of such plaque populations, especially in individuals with fatty liver. Thus, identifying carotid plaque patients in the population with fatty liver is more cost-effective and is of great public health implications for the prevention of cardiovascular disease.

Wu et al [13] developed a carotid plaque risk prediction tool among asymptomatic population based on machine learning (ML) algorithms, including extreme gradient boosting (XGBoost), gradient boosting decision tree, random forest (RF), and support vector machine, and achieved good performance, but the substantial complexity of the model may limit its practical use, while the commonly used risk prediction tools in the cardiovascular field, including Framingham risk score [15] and its modified model [16], were mostly based on traditional statistical models, including logistic regression and cox proportional-hazards regression. Although numerous studies have demonstrated that ML algorithms outperformed traditional statistical models in predictive performance throughout medical fields [17-19] due to their capability to analyze and learn the complex interactions and nonlinear associations among variables [17,20,21], the latter still own irreplaceable strengths, including their natural transparency, interpretability, and robustness, which boost their practicality in clinical research [22]. Therefore, using ML algorithms alone or traditional regression methods alone to train prediction models usually results in either accurate but complicated black boxes or practical but unsatisfactory-performed scoring systems.

In this study, we combined ML, including RF, XGBoost, and elastic net (EN) with logistic regression together to develop a straightforward and practical risk prediction model to help better identify individuals at risk of carotid plaque in the population with fatty liver disease. We also provided robust cutoff points for carotid plaque risk stratification and verified the results on an external data set.

## Methods

### Data Source and Study Participants

Participants who attended health check-ups at Meinian Health Check-up Center and MJ Health Check-up Center were involved in this study for model development and external validation, respectively.

### Development Data Set

**Figure 1:**
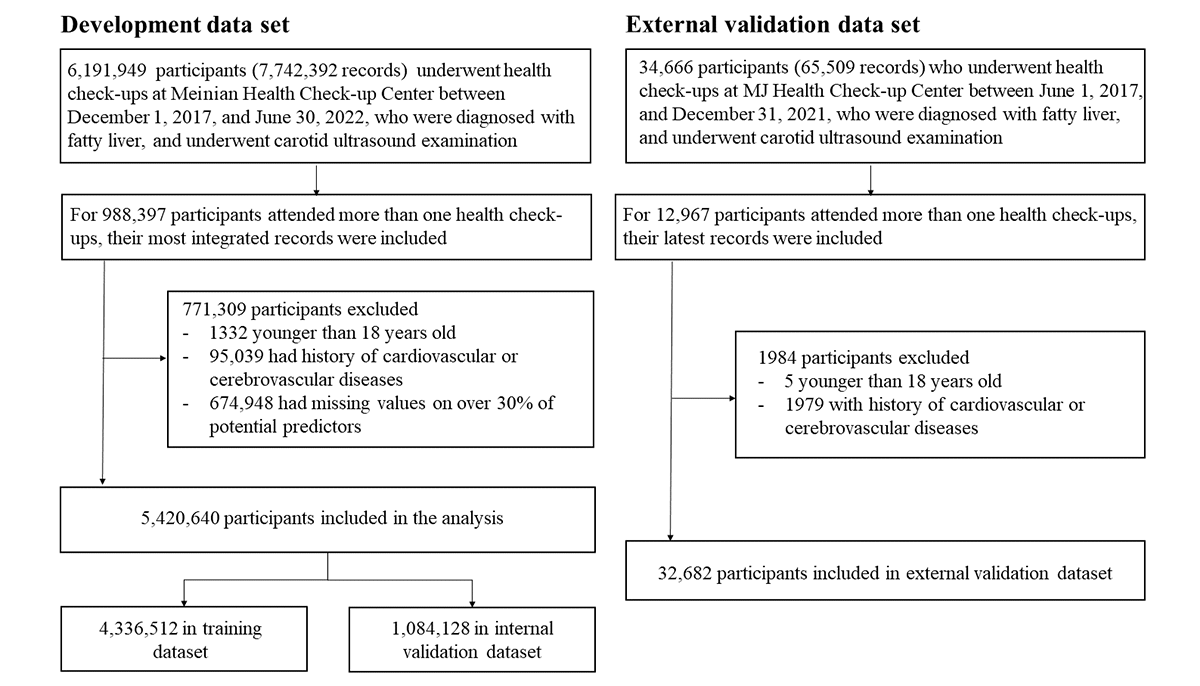
Flowchart of the study participants.

Meinian Health Check-up Program is the largest check-up chain in China. It provides routine health check-up services for the whole population, with its check-up centers covering all 31 provinces in mainland China. Initially, participants diagnosed with fatty liver through hepatic ultrasonography and received carotid artery ultrasound examination between January 1, 2017, and June 30, 2022, were included. For those who attended 2 or more check-ups, the most integrated record was included in the analysis. Participants younger than 18 years, had missing values on over 30% of potential predictors, or had a history of cardiovascular or cerebrovascular diseases were excluded from the analysis. Finally, a total of 5,420,640 participants were included in the study for model development (Figure 1).

### External Validation Data Set

MJ Health Check-up Center is a clinic in Beijing, China, it provides comprehensive health check-ups for the participants. Participants who underwent check-ups between June 1, 2017, and December 31, 2021, were diagnosed with fatty liver, and underwent carotid ultrasound examination were included. After excluding those younger than 18 years, or who had a history of cardiovascular or cerebrovascular diseases, 32,682 participants were included (Figure 1).

### Ethical Considerations

The study was reviewed and approved by the institutional review board of Peking University Health Science Center (approval ID: IRB00001052-19077). The requirement for informed consent of participants was waived due to the use of deidentified data obtained as part of routine health check-ups.

### Potential Predictors and Outcomes

All potential factors associated with carotid plaque reported by recent studies were considered. Considering the accessibility of the variables in the database, a total of 27 potential factors were extracted: (1) demographic characteristics: sex and age; (2) physical examination indicators: weight, height, BMI, systolic blood pressure (SBP), diastolic blood pressure, and heart rate; (3) laboratory examination indicators: total cholesterol (TC), triglyceride, high-density lipoprotein cholesterol, low-density lipoprotein cholesterol (LDL-C), fasting blood glucose (FBG), alanine transaminase (ALT), aspartate aminotransferase (AST), ALT/AST, direct bilirubin, total bilirubin, alkaline phosphatase (ALP), uric acid, blood platelet count (PLT), white blood cell count, creatinine, and hepatic steatosis index (HSI, which was calculated as follows: HSI=8×(ALT/AST)+BMI+2 (if diabetes mellitus)+2 (if female) [12]); and (4) medical history: hypertension, diabetes, and hyperlipidemia.

The outcome was defined as whether the participant was diagnosed with carotid plaque by carotid artery ultrasound examination. Specifically, the common carotid arteries, the bifurcation, and the external and internal carotid arteries were examined on each side by experienced sonographers operating a Doppler ultrasound system (Sonoscape S50, China) with a linear 7.5 MHz probe under standardized protocols. The distance between the leading edge of the lumen-intima echo and the leading edge of the media-adventitia echo was defined as carotid intima-media thickness. Carotid plaque was accounted as a discrete, focal wall thickening ≥1.5 mm or focal thickening >50% greater than the surrounding carotid intima-media thickness in any of the arterial segments above [23].

### Data Preprocessing and Statistical Analysis

The development data set was randomly divided into a training set (4,336,512/5,420,640, 80%) and an internal validation set (1,084,128/5,420,640, 20%). The training set was used for feature selection and model development, while the internal validation data set, together with the external validation data set, were used for model evaluation.

In the training set, missing data were imputed with the mean of each variable. And the imputed values derived from the training set were further used for missingness imputation in internal and external validation data sets. Outliers in the training set were defined as values distributed less than 1% or more than 99% quantile of the whole participants, and all the outliers were regarded as missing values.

Normally distributed continuous variables were presented as means with SDs and used the Student *t* test for statistical analysis. Non-normally distributed continuous variables were presented as a median and interquartile range and the Wilcoxon rank-sum test was used for comparison. Categorical variables were presented as counts and percentages and compared using the chi-square test.

Considering the limitations of *P* values in detecting group differences in large sample sizes, we used standardized mean difference (SMD) as an alternative method to compare the between-group differences. Unlike *P* values, SMD allows for standardized comparisons across groups despite differences in sample size, measurement scales, or variance [24]. An absolute value of SMD<0.20 can be considered as a small difference, and an absolute value of SMD<0.10 suggests a negligible difference.

### Feature Selection and Model Development

Three ML algorithms were used for feature selection, including RF, EN, and XGBoost. To tune hyperparameters in the training set, 5-fold cross-validation was conducted. The top important features coselected by 3 algorithms were used for model development.

RF and XGBoost are 2 popular ensemble learning algorithms. Both of them use decision trees to construct their models. RF generates multiple decision trees in parallel by conducting random sampling and random feature selection, and the final prediction is made by aggregating the votes from all decision trees. Feature importance in RF can be measured by evaluating the mean Gini index of each feature across multiple trees.

In contrast, XGBoost builds decision trees sequentially, where each tree is trained to correct the errors of the previous ones, and eventually, the prediction is obtained by summing the results of all trees. Feature importance in XGBoost can be quantified by calculating the average gain that a feature brings when it is chosen as the splitting variable in any decision tree.

EN model extends logistic regression by adding L1 and L2 regularization terms to overcome multicollinearity and perform feature selection. The importance of each feature in EN model can be estimated by examining the magnitude of its coefficients.

Logistic regression was used to train the final prediction model by using the features selected. Collinearity was checked through clustering analysis and the most representative feature, that is owning the smallest value of 1-R^2^ in each cluster was further selected to train logistic regression.

Model performance was assessed using discrimination and calibration. Discrimination was evaluated by area under the receiving operating characteristic curve (AUROC), and calibration was investigated through the calibration curve and Brier score. The performance of the established model was compared with a model using backward selection logistic regression without ML based-feature selection, as well as the above 3 ML models. In addition, decision curve analysis was performed to see whether the net benefit would promote when using the prediction model.

### Risk Stratification

Risk cutoff values were determined based on the Youden index, predicted risk probability distribution, and the prevalence rate of carotid plaque in the internal validation data set to divide participants into high-risk, intermediate-risk, and low-risk. Specifically, Youden index was used to identify the high-risk group, and then a cutoff value was selected for the remaining individuals based on the distribution of predicted probabilities determined by our prediction model and adjusted according to the prevalence rate of carotid plaque of the 2 groups below this cutoff point to achieve intermediate and low-risk stratification, and the effectiveness of these cutoff points was verified on an external validation data set.

All procedures were performed in SAS (version 9.4; SAS Institute) and Python (version 3.7; Python Software Foundation).

## Results

### Characteristics of Study Participants

Among the 5,420,640 participants in the development data set, 26.23% (1,421,970/5,420,640) were diagnosed with carotid plaque. The differences in all the potential predictors between participants with and without carotid plaque were statistically significant. Participants who developed carotid plaque were older, more likely to be female, and had higher SBP, diastolic blood pressure, TC, high-density lipoprotein cholesterol, LDL-C, FBG, direct bilirubin, total bilirubin, ALP, and lower height, weight, BMI, heart rate, triglyceride, ALT, AST, ALT/AST, uric acid, white blood cell count, creatinine, HSI when compared to their carotid plaque-free counterparts. The prevalence of hypertension, hyperlipidemia, and diabetes mellitus was also higher in the carotid plaque group compared to the carotid plaque-free group (Table 1). Among the 32,682 records in external validation, 21.64% (7074/32,682) were recorded for developing carotid plaque. The characteristics are presented in Table S1 of Multimedia Appendix 1. The differences between the development data set and external validation data set are presented in Table S2 of Multimedia Appendix 1.

**Table 1:** Characteristics of study participants in the development data set.

Characteristic			Total (N=5,420,640)	Carotid plaque							SMD^a^
				Yes (N=1,421,970)		No (N=3,998,670)		*P* value			
**Sex, n (%)**								<.001			–0.01
	Male	3,667,424 (67.66)		955,704 (67.21)	2,711,720 (67.82)						
	Female	1,753,216 (32.34)		466,266 (32.79)	1,286,950 (32.18)						
Age (years), mean (SD)			49.00 (39.00, 57.00)	57.00 (51.00, 64.00)		45.00 (36.00, 54.00)		<.001		1.13	
HT^b^ (cm), median (IQR)			167.00 (160.50-173.00)	166.00 (159.10-171.50)		168.00 (161.00-173.50)		<.001		–0.22	
WT^c^ (kg), mean (SD)			75.11 (12.34)	73.39 (11.40)		75.72 (12.60)		<.001		–0.19	
BMI (kg/m^2^), mean (SD)			26.90 (3.19)	26.75 (3.02)		26.96 (3.25)		<.001		–0.06	
SBP^d^ (mm Hg), mean (SD)			132.06 (18.53)	139.37 (19.67)		129.46 (17.38)		<.001		0.53	
DBP^e^ (mm Hg), mean (SD)			80.83 (12.15)	82.79 (12.22)		80.13 (12.05)		<.001		0.22	
HR^f^, (times/minute), mean (SD)			71.67 (8.35)	71.31 (8.46)		71.80 (8.31)		<.001		–0.06	
TC^g^ (mmol/L), mean (SD)			5.21 (1.03)	5.34 (1.09)		5.16 (1.00)		<.001		0.17	
TG^h^ (mmol/L), median (IQR)			1.76 (1.24-2.55)	1.76 (1.26-2.51)		1.76 (1.24-2.57)		.07		0.07	
HDL-C^i^ (mmol/L), median (IQR)			1.28 (1.10-1.44)	1.29 (1.12-1.47)		1.27 (1.09-1.43)		<.001		0.10	
LDL-C^j^ (mmol/L), mean (SD)			3.10 (0.83)	3.19 (0.87)		3.07 (0.81)		<.001		0.14	
FBG^k^ (mmol/L), median (IQR)			5.45 (4.99-6.02)	5.71 (5.19-6.52)		5.37 (4.94-5.87)		<.001		0.33	
ALT^l^ (U/L), median (IQR)			26.00 (18.30-38.50)	23.40 (17.30-33.09)		27.00 (19.00-40.32)		<.001		–0.22	
AST^m^ (U/L), median (IQR)			22.00 (18.00-27.00)	21.90 (18.00-26.30)		22.00 (18.00-27.40)		<.001		–0.05	
ALT/AST, median (IQR)			1.20 (0.94-1.52)	1.10 (0.88-1.36)		1.24 (0.97-1.58)		<.001		–0.02	
DBIL^n^ (μmol/L), mean (SD)			3.71 (1.73)	3.74 (1.83)		3.70 (1.70)		<.001		0.02	
TBIL^o^ (μmol/L), median (IQR)			13.65 (10.86-14.66)	13.65 (11.06-14.90)		13.65 (10.80-14.60)		<.001		0.04	
ALP^p^ (U/L), mean (SD)			77.86 (19.04)	79.74 (19.82)		77.19 (18.71)		<.001		0.13	
UA^q^ (μmol/L), mean (SD)			369.13 (95.66)	358.66 (91.36)		372.85 (96.87)		<.001		–0.15	
PLT^r^ (10^9^/L), mean (SD)			224.53 (57.17)	217.54 (56.48)		227.01 (57.21)		<.001		–0.17	
WBC^s^ (10^9^/L), mean (SD)			6.36 (4.74)	6.35 (5.11)		6.36 (4.60)		.01		–0.01	
Cr^t^ (μmol/L), mean (SD)			69.36 (17.10)	69.02 (17.84)		69.49 (16.82)		<.001		–0.03	
HSI^u^, mean (SD)			37.83 (98.56)	36.96 (4.63)		38.14 (114.72)		<.001		–0.01	
**Hypertension, n (%)**								<.001			0.49
	Yes	2,190,601 (40.41)		823,098 (57.88)	1,367,503 (34.20)						
	No	3,230,039 (59.59)		598,872 (42.12)	2,631,167 (65.80)						
**Hyperlipidemia, n (%)**								<.001			0.06
	Yes	2,458,476 (45.35)		676,089 (47.55)	1,782,387 (44.57)						
	No	2,962,164 (54.65)		745,881 (52.45)	2,216,283 (55.43)						
**Diabetes mellitus, n (%)**								<.001			0.34
	Yes	717,876 (13.24)		318,281 (22.38)	399,595 (9.99)						
	No	4,702,764 (86.76)		1,103,689 (77.62)	3,599,075 (90.01)						

^a^SMD: standardized mean difference.

^b^HT: height.

^c^WT: weight.

^d^SBP: systolic blood pressure.

^e^DBP: diastolic blood pressure.

^f^HR: heart rate.

^g^TC: total cholesterol.

^h^TG: triglyceride.

^i^HDL-C: high-density lipoprotein cholesterol.

^j^LDL-C: low-density lipoprotein cholesterol.

^k^FBG: fasting blood glucose.

^l^ALT: alanine transaminase.

^m^AST: aspartate aminotransferase.

^n^DBIL: direct bilirubin.

^o^TBIL: total bilirubin.

^p^ALP: alkaline phosphatase.

^q^UA: uric acid.

^r^PLT: blood platelet count.

^s^WBC: white blood cell count.

^t^Cr: creatinine.

^u^HSI: Hepatic Steatosis Index.

### Feature Importance and Model Performance

**Figure 2:**
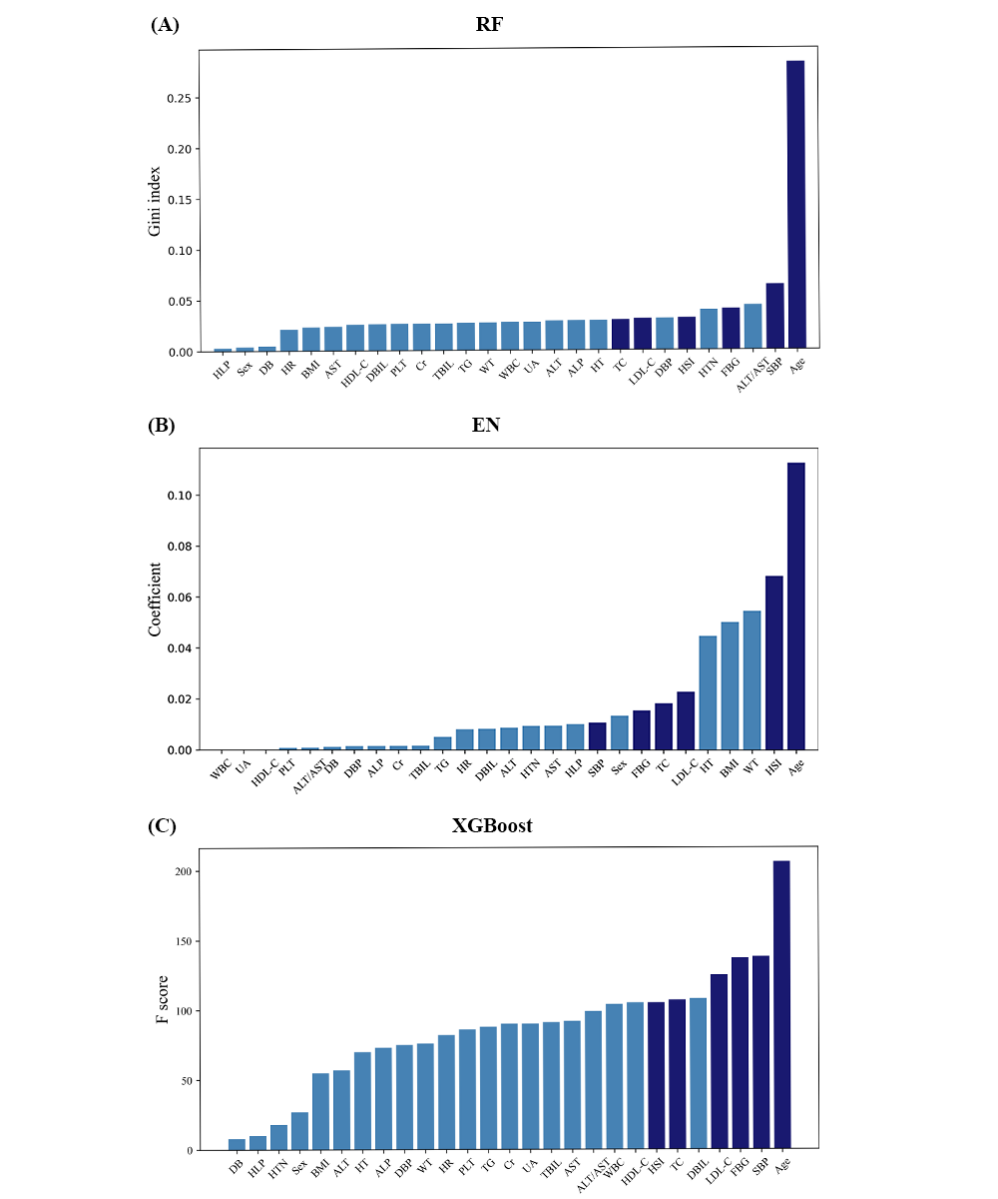
Feature importance of the potential predictors on carotid plaque in population with fatty liver disease generated by (A) RF, (B) EN, and (C) XGBoost. The features highlighted in dark color represent those coselected by all 3 algorithms. ALP: alkaline phosphatase; ALT: alanine transaminase; AST: aspartate aminotransferase; Cr: creatinine; DB: diabetes; DBIL: direct bilirubin; DBP: diastolic blood pressure; EN: elastic net; FBG: fasting blood glucose; HDL-C: high-density lipoprotein cholesterol; HLP: Hyperlipidemia; HR: heart rate; HSI: hepatic steatosis index; HT: height; HTN: hypertension; LDL-C: low-density lipoprotein cholesterol; PLT: blood platelet count; RF: random forest; SBP: systolic blood pressure; TBIL: total bilirubin; TC: total cholesterol; TG: triglyceride; UA: uric acid; WBC: white blood cell count; WT: weight; XGBoost: extreme gradient boosting.

Age, SBP, LDL-C, TC, FBG, and HSI were found to be the top important features through all 3 ML algorithms. These features ranked in the top 10 features of all 3 algorithms and were selected out of the 27 features to train the logistic regression model (Figure 2). Cluster analysis showed high collinearity existed between LDL-C and TC, thus the more informative one, LDL-C, was selected to develop the final model (Table 2). The formula for predicting the risk of carotid plaque, as determined by the final prediction model is given in the following equation:



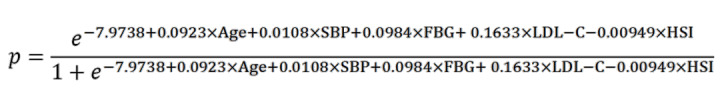



**Table 2:** Carotid plaque prediction model in population with fatty liver disease based on logistic regression.

Variable	β	SE	OR^a^ (95% CI)
Intercept	–7.97380	0.015400	
Age	0.09230	0.000133	1.097 (1.096-1.097)
SBP^b^	0.01080	0.000070	1.011 (1.011-1.011)
FBG^c^	0.09840	0.000684	1.103 (1.102-1.105)
LDL-C^d^	0.16330	0.001480	1.177 (1.174-1.181)
HSI^e^	–0.00949	0.000289	0.991 (0.990-0.991)

^a^OR: odds ratio.

^b^SBP: systolic blood pressure.

^c^FBG: fasting blood glucose.

^d^LDL-C: low-density lipoprotein cholesterol.

^e^HSI: Hepatic Steatosis Index.

**Figure 3:**
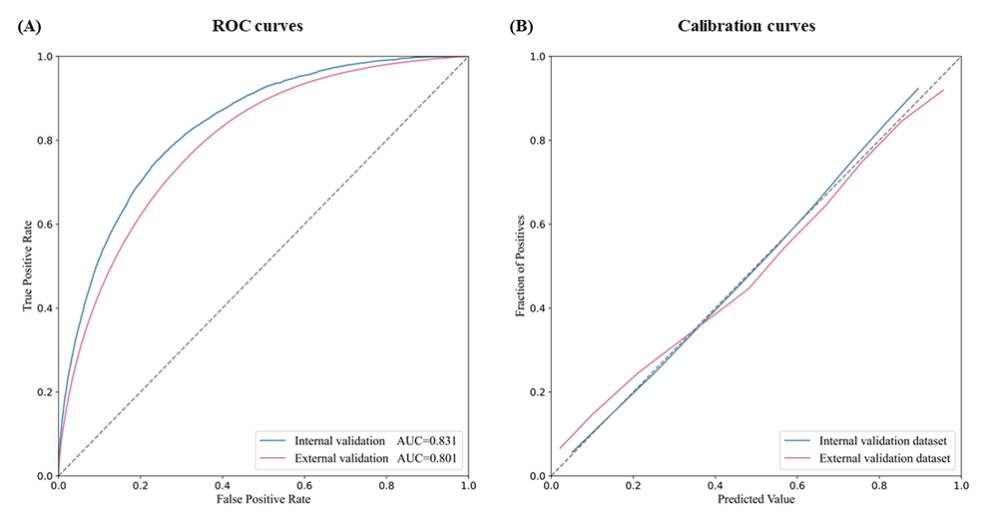
Model performance in discrimination and calibration for predicting the risk of carotid plaque in population with fatty liver disease evaluated by (A) ROC curves and (B) calibration curves. AUC: area under the curve; ROC: receiver operating characteristic.

Model performance was evaluated in internal and external validation data sets, respectively, and the area under the curves achieved 0.831 and 0.801, respectively, both showing good discrimination capability (Figure 3A). The calibration curve in the internal validation data set lies tightly against the diagonal, while the external deviates a little, but still indicates good calibration capability (Figure 3B). When compared with the backward selection logistic regression model, which consisted of 15 features, or the 3 ML models involving 27 features, the prediction model we established with only 5 features was still competitive. The number of features and model performance in each model are shown in Table 3.

**Table 3:** The comparison of model performance for predicting the risk of carotid plaques in population with fatty liver disease in the internal validation data set.

Model	Features, n	AUC^a^	Brier score
LR-ML^b^	5	0.831	0.125
LR-BS^c^	13	0.822	0.139
RF^d^	27	0.832	0.151
EN^e^	27	0.834	0.178
XGBoost^f^	27	0.831	0.150

^a^AUC: area under the curve.

^b^LR-ML: ML-based feature selection logistic regression.

^c^LR-BC: backward selection logistic regression.

^d^RF: random forest.

^e^EN: elastic net.

^f^XGBoost: extreme gradient boosting.

**Figure 4:**
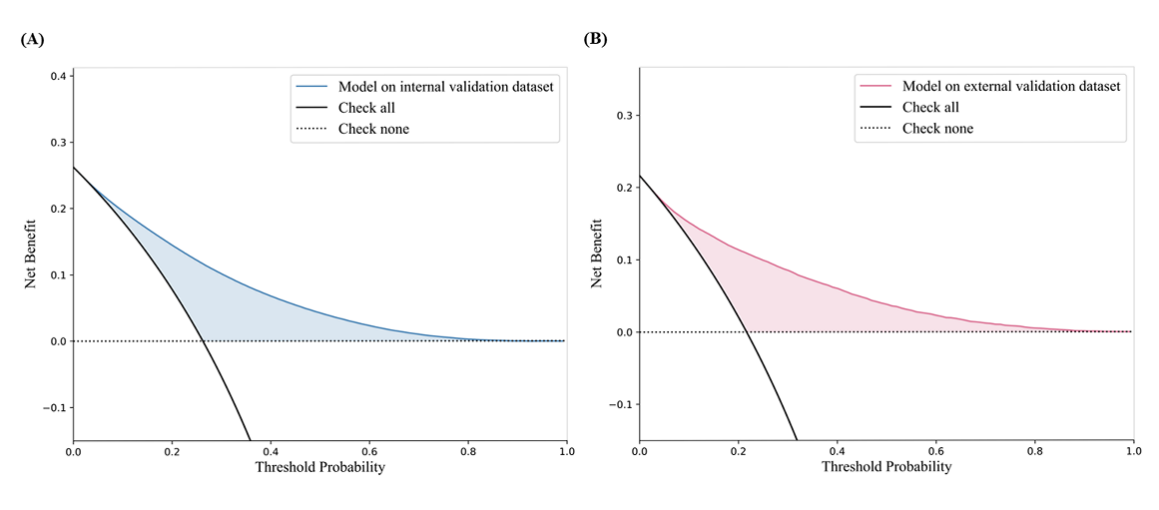
Decision curve analysis for predicting the risk of carotid plaque in population with fatty liver disease in (A) internal validation data set and (B) external validation data set.

The decision curve analysis showed that the application of the prediction model achieved promoted net benefits throughout all threshold probabilities both in internal and external validation data sets, indicating prospective utility in the real-world scenario (Figure 4).

### Risk Stratification

**Figure 5:**
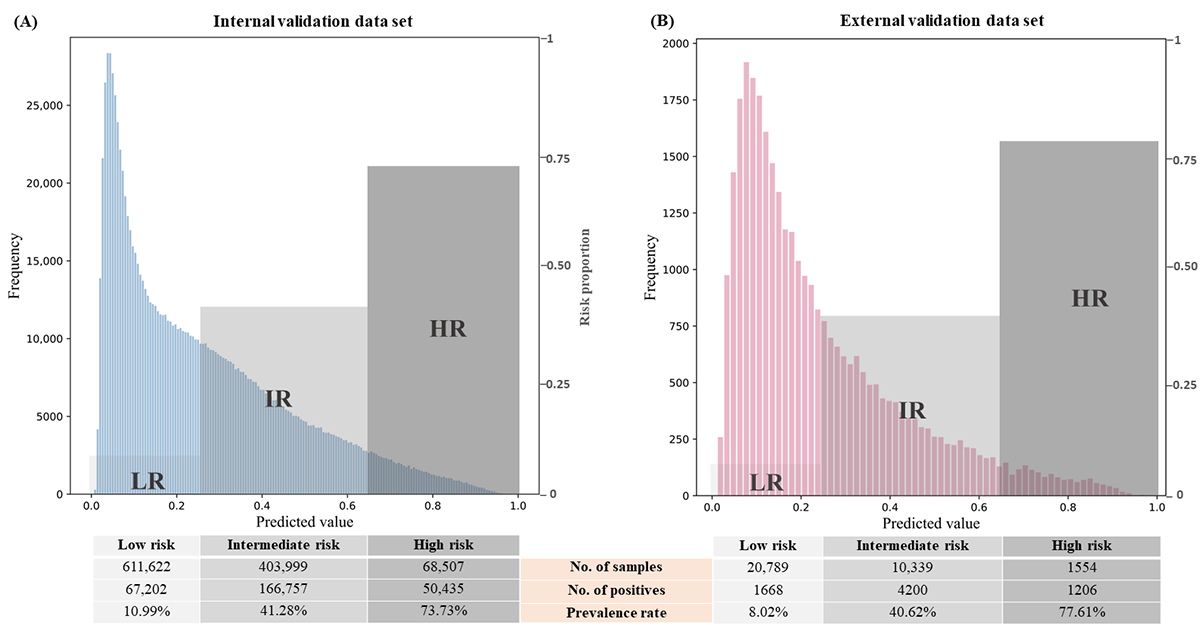
Probability distribution and risk classification plot generated by the carotid plaque prediction model in population with fatty liver disease in (A) internal validation data set and (B) external validation data set. The blue and pink colored columns represent the number of participants on different predicted probabilities, and the predicted probabilities are split into low risk, intermediate risk, and high risk by 0.25 and 0.65. Different levels of risks are presented by gray pillars of different opacities, the height of each pillar corresponds to risk proportion, which is calculated by the prevalence rate in each risk level. HR: high risk; IR: intermediate risk; LR: low risk.

The predicted risk probabilities of participants developing carotid plaque in 2 validation data sets were calculated and their histograms were presented in Figure 5. Using the Youden index, a threshold of 65% was used to categorize individuals as being at high risk, while a cutoff value of 25% was used to distinguish those at intermediate risk and low risk based on the distribution of predicted probabilities and the prevalence of carotid plaque in the remaining population. Our result revealed the prevalence of carotid plaque of 73.73%, 41.28%, and 10.99% for the high-risk, intermediate-risk, and low-risk groups, respectively. Upon application of these defined cutoff points to the external validation data set, we observed comparable prevalence rates of carotid plaque within each risk group when compared to the internal validation data set. Notably, the high-risk, intermediate-risk, and low-risk groups exhibited prevalence rates of carotid plaque amounting to 77.61%, 40.62%, and 8.02%, respectively. These findings indicate that the selected risk cutoff points can successfully stratify individuals with fatty liver disease into varying degrees of severity in terms of the risk of developing carotid plaque. The probability distribution, sample size, and prevalence rate in each level are also shown in Figure 5.

## Discussion

In this study, we established a practical and straightforward carotid plaque prediction model in population with fatty liver disease. By using only 5 features (Age, SBP, FBG, LDL-C, and HSI) coselected by 3 ML algorithms, the model achieved an AUROC of 0.831 and exhibited good calibration properties. Our study derived robust cutoff points of 25% and 65% for carotid plaque risk probability, enabling effective risk stratification and facilitating clinical decision-making regarding the need for carotid ultrasonography examination. These findings have practical implications for early detection and prevention of this condition, which can improve patient outcomes and reduce health care costs.

We identified specific features as strong predictors of the outcome. Age was selected as the top important feature by all 3 models, indicating its strong relationship with carotid plaque, prior studies have also drawn the same conclusion [8,25]. Previous research has demonstrated that increased SBP is a strong predictor of the development of carotid plaque, which was consistent with our findings [26-30]. Additionally, our study found evidence linking increased blood lipid levels, such as TC and LDL-C, and elevated glucose levels to a higher prevalence of carotid plaque. These findings are in line with prior research and support the notion that managing modifiable cardiovascular risk factors, such as dyslipidemia and hyperglycemia, is critical for reducing the likelihood of carotid plaque development [31-33]. HSI is a surrogate score for the noninvasive assessment of steatosis in patients with fatty liver [34] and is also a screening tool for nonalcoholic fatty liver disease [35]. In our study, we regarded this index as a continuous feature reflecting the severity of liver steatosis to predict carotid plaque. Although a cross-sectional study involving 768 patients with type 2 diabetes mellitus (T2DM) showed those with carotid plaque have significantly higher HSI (*P*<.001) compared with their healthy counterparts [36], our study came to the opposite conclusion. The following reason may explain the paradox. For all of the participants who have already been diagnosed with fatty liver disease, the severity may lead to behavior or lifestyle change and ulteriorly affect the development of carotid plaque. However, the lifestyle-related variables and diagnostic time of fatty liver were not included in our study, which may generate the opposite result with other studies.

Our findings revealed that the logistic regression model, comprising only 5 variables coselected by 3 ML algorithms, attained nearly equivalent area under the curve values as the ML models which included all 27 variables, but exhibited superior calibration capability. These results clearly indicated the superiority of adopting a combined approach. In regard to similar research, our predictive model remains competitive. For instance, in Wu’s [13] investigations, the XGBoost model based on 34 variables acquired an AUROC of 0.8635, whereas our model, employing solely 5 variables, yielded a comparable AUROC value of 0.831 while preserving a more comprehensible and lucid modeling framework. In practical applications, our model accurately predicts outcomes using routine, easily measurable, and obtainable variables, indicating the potential for effective clinical implementation.

We aimed to identify high-risk individuals who may benefit from carotid ultrasonography screening for carotid plaque. Therefore, it is vital not only to estimate an individual’s risk probability but also to determine optimal risk cutoff points for precise risk stratification and corresponding clinical guidance. Although the Youden index is commonly used to determine the optimal cutoff predicted probability for risk stratification, it typically results in a binary classification of high-risk and low-risk groups [37-39]. However, our results have shown that the low-risk group often comprises a larger population with a wide range of risk probabilities from 0% to 65% when performing high-low risk stratification using the Youden index alone. Although this approach can effectively identify the high-risk group, providing identical guidance to individuals within the low-risk group with significantly different risk probabilities is not appropriate and unscientific. To achieve a more comprehensive risk stratification based on our large sample data, we further stratified the low-risk group generated by the Youden index into low and intermediate risk categories using risk probability distribution and prevalence rate of carotid plaque in each group. With our substantial sample size, selecting and adjusting cutoff points based on the distribution of risk probability and prevalence rate across different strata is achievable. This novel approach enables a more nuanced risk stratification beyond the binary classification of high and low-risk groups, potentially leading to the development of personalized health care plans.

We have developed customized health care recommendations for each risk group, providing precise guidance for carotid artery ultrasound examinations. Our results indicate that individuals in the high-risk group with a prevalence of carotid plaque of over 70% should strongly consider undergoing carotid artery ultrasound for definitive diagnosis. For those in the intermediate-risk group with a prevalence of over 40%, the examination is still advised, considering their individual financial circumstances. Furthermore, low-risk individuals with a prevalence of approximately 10% do not require a carotid artery ultrasound examination. By personalizing our approach based on an individual’s risk level and financial situation, we can effectively identify those who require further testing and optimize the cost-effectiveness of screening programs.

Currently, there are no established criteria for determining which populations require carotid ultrasound screening. Several guidelines and recommendations have been proposed to identify populations that may benefit from carotid ultrasound screening. For example, the guidelines for carotid artery ultrasound examination in Chinese health check-up populations specify that the evaluation standards for individuals undergoing carotid artery ultrasound include those at risk of hypertension, coronary atherosclerotic heart disease, stroke, and diabetes; high-risk populations such as smokers, overweight and obese individuals; individuals with moderate or higher cardiovascular risk assessment; and other suitable populations aged middle-aged or older. The American Heart Association recommends carotid ultrasound screening for asymptomatic patients who are over 65 years of age, men aged 55 to 75 years with a history of smoking or other risk factors, and women aged 55 to 75 years with a history of cardiovascular disease or other risk factors. However, the existing guidelines are primarily focused on risk stratification at the population level. Our objective, therefore, is to develop a prediction model for personalized risk stratification to enable better decision-making support in determining the need for carotid artery ultrasound monitoring on an individual basis. This approach would lead to more precise and personalized health care recommendations for the individuals under consideration.

Our prediction model and the cutoff points were verified on an independent external data set. The model was also able to accurately predict the risk of carotid plaque for each individual and the cutoff points remain robust in identifying different risk levels of groups, confirming the generalizability and applicability of our approach.

Several limitations need to be noted. First, due to the limitation of the database, some lifestyle variables, like smoking or drinking status [40,41], were not included in our model, which may affect the predictive performance to some extent. Second, because of the high calculation time cost caused by the huge sample size, bootstrap sampling was not used to generate a 95% CI of the performance metrics, a single measurement may not be forceful enough. Third, we opted to exclude individuals with cardiovascular and cerebrovascular diseases from our study sample, in recognition of their potential differences in baseline characteristics, health care–seeking behavior, and management strategies relative to the general population. These factors could introduce significant confounding effects and hinder the predictive performance of our model. Therefore, we excluded individuals with cardiovascular and cerebrovascular diseases at the beginning. However, this may have reduced the representativeness of our sample and introduced some selection bias. Fourth, although the check-up centers included in this study covered all provinces and all 3 economic zones (the eastern zone, central zone, and western zone) in mainland China, the check-up population may not be entirely representative of the general population, which may have biased our study towards a healthier group and thus limit the applicability and generalizability of our model to the broader population. Fifth, the cross-sectional design of our study means that the temporal relationship between the predictors and the outcome cannot be established, and there may be reverse causation or confounding effects that we have not accounted for.

In conclusion, we developed a prediction model that uses a set of routine and quantitative variables obtained from health checkup programs to estimate the risk of carotid plaque in individuals with fatty liver disease. The resulting model is cost-effective, easy to use, and demonstrated strong predictive performance. This approach provides a means for personalized risk assessment of carotid plaque and derives robust cutoff points for carotid plaque risk stratification, with potential implications for improving the cost-effectiveness of carotid ultrasound detection.

## Data Availability

The data are not publicly available as they are individual-level health check-up data**,** but are available from the corresponding author on reasonable request.

## Appendix

Multimedia Appendix 1 Characteristics of study participants in the external validation data set or characteristics of study participants in the development data set and external validation data set.

## Abbreviations

ALP: alkaline phosphatase
ALT: alanine transaminase
AST: aspartate aminotransferase
AUROC: area under the receiver operating characteristic curve
EN: elastic net
FBG: fasting blood glucose
HSI: Hepatic Steatosis Index
LDL-C: low-density lipoprotein cholesterol
ML: machine learning
PLT: blood platelet count
RF: random forest
SBP: systolic blood pressure
SMD: standardized mean difference
T2DM: type 2 diabetes mellitus
TC: total cholesterol
XGBoost: extreme gradient boosting
