# Supplementary material for: Combinatorial Use of Machine Learning and Logistic Regression for Predicting Carotid Plaque Risk Among 5.4 Million Adults With Fatty Liver Disease Receiving Health Check-Ups: Population-Based Cross-Sectional Study
Source: JMIR Public Health Surveill. 2023 Sep 7;9:e47095. doi: 10.2196/47095 (PMC10514774; doi:10.2196/47095)
Supplement: Multimedia Appendix 1 [file publichealth_v9i1e47095_app1.docx]

**Multimedia Appendix 1**

**Table S1.** Characteristics of study participants in the external validation dataset.

| Characteristic, n (%) | | Total (N=32,682) | Carotid Plaque | | |  |
| --- | --- | --- | --- | --- | --- | --- |
|  |  |  | Yes (N=7074) | No (N=25,608) | *P* value | SMD^a^ |
|  | |  |  |  |  |  |
| Sex | |  |  |  | <.001 | –0.09 |
|  | Male | 24,397 (74.65) | 5067 (71.63) | 19,330 (75.48) |  |  |
|  | Female | 8285 (25.35) | 2007 (28.37) | 6278 (24.52) |  |  |
| Age, years | | 48.00 (41.00, 56.00) | 58.00 (51.00, 65.00) | 45.00 (39.00, 52.00) | <.001 | 1.31 |
| HT^b^, cm | | 170.00 (164.10, 175.10) | 168.70 (162.20, 173.70) | 170.50 (164.80, 175.60) | <.001 | –0.27 |
| WT^c^, kg | | 76.91 (11.80) | 75.13 (11.53) | 77.40 (11.83) | <.001 | –0.19 |
| BMI, kg/m^2^ | | 26.70 (3.07) | 26.62 (3.01) | 26.72 (3.09) | .009 | –0.03 |
| SBP^d^, mm Hg | | 122.26 (15.85) | 128.61 (17.24) | 120.51 (14.98) | <.001 | 0.50 |
| DBP^e^, mm Hg | | 76.33 (11.05) | 77.82 (11.06) | 75.92 (11.02) | <.001 | 0.17 |
| HR^f^, times/min | |  |  |  |  |  |
| TC^g^, mmol/L | | 4.93 (0.93) | 4.95 (1.00) | 4.92 (0.91) | .03 | 0.03 |
| TG^h^, mmol/L | | 1.65 (1.20, 2.31) | 1.66 (1.23, 2.29) | 1.64 (1.19, 2.31) | .51 | 0.01 |
| HDL-C^i^, mmol/L | | 1.17 (1.01, 1.36) | 1.18 (1.02, 1.38) | 1.16 (1.00, 1.35) | <.001 | 0.09 |
| LDL-C^j^, mmol/L | | 3.24 (0.85) | 3.30 (0.91) | 3.22 (0.83) | <.001 | 0.10 |
| FBG^k^, mmol/L | | 5.72 (5.40, 6.20) | 6.00 (5.60, 6.80) | 5.70 (5.40, 6.10) | <.001 | 0.39 |
| ALT^l^, U/L | | 24.00 (18.00, 35.00) | 22.00 (16.00, 31.00) | 25.00 (18.00, 37.00) | <.001 | –0.19 |
| AST^m^, U/L | | 20.00 (17.00, 24.00) | 20.00 (16.00, 24.00) | 20.00 (17.00, 25.00) | <.001 | <.001 |
| ALT/AST | | 1.30 (0.43) | 1.18 (0.38) | 1.33 (0.44) | <.001 | –0.36 |
| DBIL^n^, μmol/L | | 3.98 (3.00, 5.00) | 4.18 (3.34, 5.28) | 3.90 (2.92, 4.92) | <.001 | 0.26 |
| TBIL^o^, μmol/L | | 11.30 (8.30, 14.80) | 11.80 (9.00, 15.20) | 11.20 (8.10, 14.70) | <.001 | 0.15 |
| ALP^p^, U/L | | 70.24 (18.32) | 72.35 (20.05) | 69.65 (17.77) | <.001 | 0.14 |
| UA^q^, μmol/L | | 368.19 (83.65) | 360.91 (81.41) | 370.20 (84.15) | <.001 | –0.11 |
| PLT^r^, 10^9^/L | | 227.37 (54.36) | 219.07 (54.19) | 229.66 (54.19) | <.001 | –0.20 |
| WBC^s^, 10^9^/L | | 6.00 (5.20, 7.10) | 6.00 (5.10, 7.10) | 6.10 (5.20, 7.10) | .02 | –0.02 |
| Cr^t^, μmol/L | | 74.00 (62.00, 84.00) | 75.00 (64.00, 85.00) | 74.00 (61.00, 84.00) | <.001 | 0.23 |
| HSI^u^ | | 37.76 (3.96) | 37.15 (3.33) | 37.92 (4.11) | <.001 | –0.21 |
| Hypertension | |  |  |  | <.001 | 0.56 |
|  | Yes | 10,083 (30.85) | 3641 (51.47) | 6442 (25.16) |  |  |
|  | No | 22,599 (69.15) | 3433 (48.53) | 19,166 (74.84) |  |  |
| Hyperlipidemia | |  |  |  | <.001 | 0.18 |
|  | Yes | 16,176 (49.50) | 4004 (56.60) | 12,172 (47.53) |  |  |
|  | No | 16,506 (50.50) | 3070 (43.40) | 13,436 (52.47) |  |  |
| Diabetes mellitus | |  |  |  | <.001 | 0.45 |
|  | Yes | 5183 (15.86) | 2111 (29.84) | 3072 (12.00) |  |  |
|  | No | 27,499 (84.14) | 4963 (70.16) | 22,536 (88.00) |  |  |

^a^SMD: standardized mean difference.

^b^HT: height.

^c^WT: weight.

^d^SBP: systolic blood pressure.

^e^DBP: diastolic blood pressure.

^f^HR: heart rate.

^g^TC: total cholesterol.

^h^TG: triglyceride.

^i^HDL-C: high-density lipoprotein cholesterol.

^j^LDL-C: low-density lipoprotein cholesterol.

^k^FBG: fasting blood glucose.

^l^ALT: alanine transaminase.

^m^AST: aspartate aminotransferase.

^n^DBIL: direct bilirubin.

^o^TBIL: total bilirubin.

^p^ALP: alkaline phosphatase.

^q^UA: uric acid.

^r^PLT: blood platelet count.

^s^WBC: white blood cell count.

^t^Cr: creatinine.

^u^HSI: Hepatic Steatosis Index.

**Table S2**. Characteristics of study participants in the development dataset and external validation dataset.

| Characteristic, n (%) | | Total (N=5,453,322) | Dataset | | |  |
| --- | --- | --- | --- | --- | --- | --- |
|  |  |  | Development (N=5,420,640) | External validation (N=32,682) | *P* value | SMD^a^ |
|  | |  |  |  |  |  |
| Sex | |  |  |  | <.001 | –0.15 |
|  | Male | 3,691,821 (67.70) | 3,667,424 (67.66) | 24,397 (74.65) |  |  |
|  | Female | 1,761,501 (32.30) | 1,753,216 (32.34) | 8285 (25.35) |  |  |
| Age, years | | 49.00 (39.00, 57.00) | 49.00 (39.00, 57.00) | 48.00 (41.00, 56.00) | .04 | 0.04 |
| HT^b^, cm | | 167.00 (160.50, 173.00) | 167.00 (160.50, 173.00) | 170.00 (164.10, 175.10) | <.001 | –0.32 |
| WT^c^, kg | | 75.12 (12.34) | 75.11 (12.34) | 76.91 (11.80) | <.001 | –0.15 |
| BMI, kg/m^2^ | | 26.90 (3.19) | 26.90 (3.19) | 26.70 (3.07) | <.001 | 0.06 |
| SBP^d^, mm Hg | | 132.00 (18.53) | 132.06 (18.53) | 122.26 (15.85) | <.001 | 0.57 |
| DBP^e^, mm Hg | | 80.80 (12.15) | 80.83 (12.15) | 76.33 (11.05) | <.001 | 0.39 |
| HR^f^, times/min | |  |  |  |  |  |
| TC^g^, mmol/L | | 5.21 (1.03) | 5.21 (1.03) | 4.93 (0.93) | <.001 | 0.29 |
| TG^h^, mmol/L | | 1.76 (1.24-2.55) | 1.76 (1.24-2.55) | 1.65 (1.20-2.31) | <.001 | 0.13 |
| HDL-C^i^, mmol/L | | 1.28 (1.10-1.44) | 1.28 (1.10-1.44) | 1.17 (1.01-1.36) | <.001 | 0.29 |
| LDL-C^j^, mmol/L | | 3.10 (0.83) | 3.10 (0.83) | 3.24 (0.85) | <.001 | –0.17 |
| FBG^k^, mmol/L | | 5.45 (5.00, 6.02) | 5.45 (4.99, 6.02) | 5.72 (5.40, 6.20) | <.001 | –0.19 |
| ALT^l^, U/L | | 26.00 (18.30, 38.50) | 26.00 (18.30, 38.50) | 24.00 (18.00, 35.00) | <.001 | 0.11 |
| AST^m^, U/L | | 22.00 (18.00, 27.00) | 22.00 (18.00, 27.00) | 20.00 (17.00, 24.00) | <.001 | 0.18 |
| ALT/AST | | 1.20 (0.94, 1.52) | 1.20 (0.94, 1.52) | 1.24 (1.00, 1.54) | <.001 | –0.01 |
| DBIL^n^, μmol/L | | 3.71 (2.70, 4.10) | 3.71 (2.70, 4.10) | 3.98 (3.00, 5.00) | <.001 | –0.17 |
| TBIL^o^, μmol/L | | 13.65 (10.81, 14.66) | 13.65 (10.86, 14.66) | 11.30 (8.30, 14.80) | <.001 | 0.32 |
| ALP^p^, U/L | | 77.81 (19.05) | 77.86 (19.04) | 70.24 (18.32) | <.001 | 0.41 |
| UA^q^, μmol/L | | 369.12 (95.59) | 369.13 (95.66) | 368.19 (83.65) | .04 | 0.01 |
| PLT^r^, 10^9^/L | | 224.54 (57.16) | 224.53 (57.17) | 227.37 (54.36) | <.001 | –0.05 |
| WBC^s^, 10^9^/L | | 6.20 (5.26, 7.19) | 6.20 (5.26, 7.19) | 6.00 (5.20, 7.10) | <.001 | 0.03 |
| Cr^t^, μmol/L | | 69.37 (17.15) | 69.36 (17.10) | 69.86 (24.92) | <.001 | –0.02 |
| HSI^u^ | | 37.83 (34.57, 40.19) | 37.83 (34.57, 40.20) | 37.76 (36.09, 38.38) | <.001 | <.001 |
| Hypertension | |  |  |  | <.001 | 0.20 |
|  | Yes | 2,200,684 (40.35) | 2,190,601 (40.41) | 10,083 (30.85) |  |  |
|  | No | 3,252,638 (59.65) | 3,230,039 (59.59) | 22,599 (69.15) |  |  |
| Hyperlipidemia | |  |  |  | <.001 | –0.08 |
|  | Yes | 2,474,652 (45.38) | 2,458,476 (45.35) | 16,176 (49.50) |  |  |
|  | No | 2,978,670 (54.62) | 2,962,164 (54.65) | 16,506 (50.50) |  |  |
| Diabetes mellitus | |  |  |  | <.001 | –0.07 |
|  | Yes | 723,059 (13.26) | 717,876 (13.24) | 5183 (15.86) |  |  |
|  | No | 4,730,263 (86.74) | 4,702,764 (86.76) | 27,499 (84.14) |  |  |

^a^SMD: standardized mean difference.

^b^HT: height.

^c^WT: weight.

^d^SBP: systolic blood pressure.

^e^DBP: diastolic blood pressure.

^f^HR: heart rate.

^g^TC: total cholesterol.

^h^TG: triglyceride.

^i^HDL-C: high-density lipoprotein cholesterol.

^j^LDL-C: low-density lipoprotein cholesterol.

^k^FBG: fasting blood glucose.

^l^ALT: alanine transaminase.

^m^AST: aspartate aminotransferase.

^n^DBIL: direct bilirubin.

^o^TBIL: total bilirubin.

^p^ALP: alkaline phosphatase.

^q^UA: uric acid.

^r^PLT: blood platelet count.

^s^WBC: white blood cell count.

^t^Cr: creatinine.

^u^HSI: Hepatic Steatosis Index.
